# Prevalence of stress among medical students: a comparative study between public and private medical schools in Bangladesh

**DOI:** 10.1186/s13104-015-1295-5

**Published:** 2015-07-30

**Authors:** Eliza Omar Eva, Md Zakirul Islam, Abu Syed Md Mosaddek, Md Faizur Rahman, Rini Juliet Rozario, A F Md Hassan Iftekhar, Tarafder Shahniam Ahmed, Iffat Jahan, Abdullahi Rabiu Abubakar, Wan Putri Elena Wan Dali, Mohammed S Razzaque, Rahat Bin Habib, Mainul Haque

**Affiliations:** Department of Pharmacology and Therapeutics, Dhaka Medical College, Dhaka, 1000 Bangladesh; Department of Pharmacology and Therapeutics, Eastern Medical College, Comilla, 3520 Bangladesh; Department of Pharmacology and Therapeutics, Uttara Adhunik Medical College, Dhaka, 1230 Bangladesh; Department of Pharmacology and Therapeutics, Shaheed Monsur Ali Medical College, Dhaka, 1230 Bangladesh; Department of Pharmacology and Therapeutics, Central Medical College, Comilla, 3503 Bangladesh; Department of General and Dental Pharmacology, Sapporo Dental College and General Hospital, Dhaka, 1230 Bangladesh; Department of Physiology, Chittagong Medical College, Chittagong, 4218 Bangladesh; Unit of Pharmacology, Faculty of Medicine, Universiti Sultan Zainal Abidin, Medical Kampus, Jalan Sultan Mahmud, 20400 Kuala Terengganu, Terengganu Malaysia; School of Health Sciences, Health Campus, Universiti Sains Malaysia, 16150 Kubang Kerian, Kelantan Malaysia; Department of Oral Medicine, Harvard School of Dental Medicine, 190 Longwood Avenue, Boston, MA 02115 USA; Sir Salimullah College and Hospital, Dhaka, 1000 Bangladesh

**Keywords:** Stress, Medical-students, Bangladesh

## Abstract

**Background:**

Throughout the world all health professionals face stress because of time-pressures, workload, multiple roles and emotional issues. Stress does not only exist among the health professionals but also in medical students. Bangladesh has currently 77 medical colleges 54 of which are private. This study was designed to collect baseline data of stress-level among Bangladeshi students, which we believe will form the basis for further in depth studies.

**Methods:**

A cross-sectional study was conducted on medical students from 2 public and 6 private medical-schools in Bangladesh. All medical schools have common curriculum formulated by the Government of Bangladesh. The study population was 1,363 medical students of Year-III and IV of academic session 2013/2014. Universal sampling technique was used. The period of study was February to June 2014. Data was collected using a validated instrument, compiled and analysed using SPSS version-20.

**Results:**

A total of 990 (73%) out 1,363 medical students participated in the study, of which 36% were male and 64% were female. The overall prevalence of stress of the study population was 54%. 53% of male and 55% of female were reported suffering from stress. 54% of Year-III students and 55% of Year-IV were noted suffering from stress. There was statistically significant (p = 0.005) differences in the level of stress between public (2.84 ± 0.59) and private (2.73 ± 0.57) medical schools student.

**Conclusions:**

More than half of Bangladeshi medical students are suffering from measureable academic stress. It would be pertinent if the relevant authorities could address the issue so as to provide a conducive medical learning environment.

**Electronic supplementary material:**

The online version of this article (doi:10.1186/s13104-015-1295-5) contains supplementary material, which is available to authorized users.

## Background

Worldwide, medical colleges are responsible for making sure that medical students have adequate knowledge and skill before taking professional responsibilities [1]. In order to achieve these goals, medical colleges typically use a curriculum of lectures, simulations supervised practice, mentoring, and hands-on experience to boost individual skill-set. Unfortunately, some aspects of the training process have unintended negative consequences on students’ physical and emotional health [1, 2]. Studies revealed that medical students experience a relatively high level of personal distress, with adverse consequences on academic performance, competency, professionalism, and health [2–5]. It is imperative that medical college educator understand the incidences and causes of student distress, adverse consequences on personal and professional well-being, and institutional factors that has an impact on students health [6]. Stress experienced by medical students start from the beginning of the training process [3]. Although some degree of stress is accepted as a normal part of medical training and can be a motivator for some individuals, not all students find the stress manageable [3–5]. Stress may give rise to feelings of fear, incompetence, uselessness, anger, and guilt and has been associated with both psychological and physical disorders [6]. Medical students have used various coping mechanisms to deal with stress; the coping strategies applied by students may determine the effect of stress on psychological and physical health and may determine whether stress has a positive or negative influence. Ineffective stress coping mechanism such as problem avoidance, cynical thinking, social withdrawal, and self-criticism, has negative consequences and can lead to depression, anxiety, and poor mental health. However, appropriate strategies such as problem solving, positive interpretation, and social support can enable students to respond in a manner that leads to adaptation [6].

Medical education is generally perceived as being stressful. High rates of psychological morbidity among students, such as anxiety and depressive symptoms, have been reported in several studies [2, 6–8]. Various measures have been used to address these phenomena which are directed towards specific medical school stressors such as burn-out or general aspects of stress. Depressive symptomatology in medical students has been assessed with Beck’s Depression Inventory (BDI), the 12-item General Health Questionnaire (GHQ-12) and different versions of the Symptom Check-List (SCL) [9–12]. Several studies were conducted to examine the sources of stress among medical students and these are based on three main areas: academic pressures, social issues and financial difficulties [13]. The majority of stressful conditions were found to be related to medical training rather than personal problems. Depression and anxiety were found to be associated with workload, fears of failing, personal endurance, and lack of time for other activities [13]. Research has shown that Canadian medical students experience less stress than law students, postgraduate students and the general population [14]. In a longitudinal study among UK medical students, Year 1 students were found to have the highest levels of mental distress hence they are most likely to have problem in subsequent years [15]. Medical students in the University of Massachusetts have shown increased stress and depression rates in their 2nd and 4th year [16]. In a study on gender differences in the University of São Paolo, female students were found to be more affected by both depression and anxiety than males [8].

A number of stressors are associated with the health professions, including time pressures, workload, having multiple roles, and emotional issues [17]. Frequent exposure to environmental stress associated with human pain and distress in the workplace can have an impact on the physical and mental wellbeing of health professionals and result in burnout and, in some cases, traumatic stress-like symptoms. These negative stress outcomes can affect not only the well-being of health professionals, but also on their ability to care effectively for others [17]. Developing and fostering resilient environments within the health profession is of paramount importance. Production of resilient health care professionals with conducive and well-equipped hospitals is emerging as a way to reduce negative, and increase positive outcomes of stress in health profession [17]. The definition of ‘resilience’ has been adapted from the developmental psychology literature as the ability to maintain personal and professional wellbeing in the face of on-going work stress and adversity [17].

### What is stress?

Stress is the “wear and tear” our bodies experience as we adjust to our continually changing environment; it has physical and emotional effects and can create positive or negative influence on us [18]. As a positive influence, stress can help to compel us for action. As a negative influence, it can result in feelings of distress, rejection, anger, and depression, which in turn can lead to health problems [18]. Medically, stress is when a person feels threatened by or under pressure from a particular situation and their body reacts accordingly. Hormones are released to prepare the body for action. The heartbeat increases and blood pressure rises. More blood flow to the heart and the major muscles. Flow is diverted away from “less important” areas such as the digestive system. Nausea is often experienced during stress [18]. Other illness believed to be caused by stress includes angina, asthma, cancer, cystitis, depression, diabetes, diarrhoea, heart attack, migraine, psoriasis rheumatoid arthritis, and ulcers. Partial loss of body hair (alopecia areata) or even total loss of all body hair (alopecia universalis) can also result from stress [18].

### Is stress necessary?

Naturally everyone needs a certain amount of “pressure” to perform at their best. But when pressures exceed a person’s ability to cope, the result is stress. Prolonged stress can set up distress and shut down the ability to cope with ordinary situations causing illnesses [18].

Stress during medical school can lead to problems later in professional life compromising patient care [19]. Stress has been reportedly associated with anxiety, depression and psychological symptoms plausibly having a negative impact on student’s academic performance [20, 21]. Stress in medical students has been a global issue [22]. As Bangladesh is a developing country, thus there are strong possibilities that their medical student has a higher level of stress. A study conducted 5–6 years before reported stress levels in medical students of Bangladesh [23]. There was no multicentre comparative study between private and public medical schools done. This study was done with the intention to provide baseline data for future studies and for Bangladeshi authority to take initiative to safeguard the wellbeing of the future Bangladeshi medical doctors.

### Study objectives

(a) To evaluate medical students stress indicators. (b) To assess the cause of medical students’ stress. (c) To evaluate medical students stress coping mechanism.

## Methods

This is a cross-sectional study conducted among medical students of eight medical schools of Bangladesh selected purposively based on feasibility and interest of faculties in the study. The participating Medical schools are Dhaka Medical College (DMC), Sir Salimullah Medical College (SSMC), Eastern Medical College (EMC), Gazi Medical College (GMC), Central Medical College (CMC), ZH Sikder Medical College (ZHSMC), Uttara Adhunik Medical College (UAMC), and Sahid Monsur Ali Medical College (SMAMC). DMC and SSMC are public medical schools and the rests are private institutions. All cited medical schools are institutions of public universities. These schools follow common curriculum formulated by the Government of Bangladesh. A summary of the curriculum is described in Additional file 1: Table S1 [24]. Very recently the curriculum was again updated. The study participants were following the earlier curriculum (Additional file 1: Table S1) [24]. The study population was 1,363 medical students from Year-III and IV during the academic session 2013/2014. Convenient cluster sampling was used in which the whole students of Year III and IV who were willing to patriciate were included. The period of study was from February to June 2014. The instrument developed and validated by Yusoff et al. [25] was adopted for use. The questionnaire was pretested again and validated for Bangladeshi context. The questionnaire comprised of four parts. Section I: Socio-Demographic Characteristics; Section II: General Health Question (GHQ-12); Section III: The Medical Student Stressor Questionnaire; and Section IV: Coping Strategies. The questionnaire can be found in Additional file 2.

An extensively used instrument to quantify stress levels is the 12-item General Health Questionnaire (GHQ-12). A number of studies have validated the consistency GHQ-12 coefficients to be ranging from 0.78 to 0.95. The items on the GHQ-12 epitomise the 12 manifestations of stress and respondents were required to rate the occurrence of each of these manifestations in themselves just before the study period. Subjects responded to each question by choosing from four typical responses: ‘not at all’, ‘no more than usual’, ‘rather more than usual’ and ‘much more than usual’. A binary recording scheme is used to appraise the answers. This technique allocates a score of 0 to the two least symptomatic replies and a score of 1 to the two most symptomatic answers; thus, responses can only be scored as 0 or 1 [25–27].

A newly developed instrument, the Medical Students Stressor Questionnaire (MSSQ), was used to identify the source of stress. The items on MSSQ represent 40 events that have been identified to be the most probable source of stress in medical students. Respondents were requested to assess each event in them during the recent weeks by choosing from five responses: ‘causing no stress at all’, ‘causing mild stresses, ‘causing moderate stress’, ‘causing high stress’ and ‘causing severe stress’. The MSSQ is scored by assigning a value of 0–4 for each of the respective responses. A response of ‘causing no stress at all’ would be scored as 0 and a response of ‘causing severe stresses’ scored as 4 [25–27].

The questionnaires were semi-structured, self-administered which were distributed to the Year-III and IV medical students during a meeting in the lecture hall. The process of filling in the questionnaire took about 15 min. This questionnaire based study was anonymous and medical students’ participation was entirely voluntarily. The data was then compiled and analysed using SPSS version-20. Descriptive statistics was carried out; Independent *t* test was performed to find the mean differences between stress indicators. This study obtained ethical approval from Dhaka Medical College, Dhaka, Bangladesh; Memo No. MEU-DMC/ECC/2014/51 Dated: 04/08/2014. All the study respondents were verbally explained about study objectives, about animosity, and research ethics. As the study was based on questionnaire therefore Medical colleges’ authority has given verbal permission to collect the data pending the ethical clearance certificate.

## Results

From a total of 1,363 medical students, 990 filled and returned the questionnaire giving the response rate of 73%, with following distribution: DMC (n = 249), SSMC (n = 88), EMC (n = 134), GMC (n = 103), CMC (n = 84), ZHSMC (n = 116), UAMC (n = 130). Males were 36% (357) and females were 64% (633). Year-III medical students were 58% (579) and Year-IV medical students were 42% (411). A majority 83.2% (824) professed being Muslim, 16.2% (160) were Hindu, 0.2% (2) were Christian and 0.4% (4) were others. Ninety-eight percent (968) of students were single but only 2% (22) were married. In addition, 64% (636) of the students live in hostel, 26% (255) live with parents’ and 10% (99) in rental home (Additional file 1: Table S2). Similarly, 65% (646) of the students were admitted to medical school by own motivation and interest, 22% (220) due to parenteral influence and 13% (124) out of random choice. Average age of the study population was 21.52 ± 1.16 years. During the study period, the medical students were posted to medicine, surgery, O and G, paediatrics, orthopaedics, psychiatry, skin and VD, ophthalmology, otolaryngology, radiology, anaesthesiology, etc.

Out of 1,363 students, 49% (488) felt that their concentration is the same as before; 62% (616) indicated that they has a useful role in medical school; 55% (547) felt they are capable of making good decision. Similarly, 41% (408) reported that they enjoyed their daily activities; 50% (499) were able to confront their problems. In addition, 55% (540) were reasonably happy; 32% (319) do not lose their sleep due to anxieties. Meanwhile, 32% (313) felt they were constantly under stress; 37% (362) could overcome their difficulties. In contrast, 26% (260) felt unhappy and depressed; 38% (380) were not losing confidence; 52% (518) do not felt they are worthless (Additional file 1: Table S3).

The overall prevalence of stress GHQ scores of 4 as the cut-off point among the study population was 54%. Out of these, 53% (189) male and 55% (345) female were suffering from stress. Also, 54% (310) of Year-III medical students and 55% (224) of Year-IV medical students were suffering from stress. The mean ± SD of positive item GHQ-12 were 2.74 ± 0.53 and negative items 2.79 ± 0.74 (Additional file 1: Table S3) [28]. The status of each stressor based on the degree of stress experienced by the students was illustrated (Additional file 1: Table S3). Academically linked problems are primary reasons of stress among Bangladeshi medical students (Additional file 1: Table S4). This study is not able to find any other issues like gender; religion, etc. that are responsible for stress among the medical students.

There was significant (p = 0.005) differences between public (2.84 ± 0.59) and private (2.73 ± 0.57) medical schools for GHQ scores of the study participants (Additional file 1: Table S5). Similarly, significant (p = 0.004) differences were also observed between Year-III (2.81 ± 0.58) and IV (2.70 ± 0.58) (Additional file 1: Table S5). However, there was no significant (p = 0.060) differences observed between male (2.81 ± 0.57) and female (2.74 ± 0.59) (Additional file 1: Table S5) respondents.

Study participants reported 15 varieties of coping strategies for minimizing their stress. Those are: (1) share with friend and family (135, 14%); (2) watch TV and movie (126, 13%); (3) self-counselling to think positively and try hard to do the best (123, 12%); (4) say prayers (117, 12%); (5) taking rest and sleep (92, 9%); (6) singing and listening songs (90, 9%); (7) net surfing (88, 9%); (8) reading story books and diary writing (69, 7%); (9) playing indoor and outdoor games (42, 4%); (10) solitary and silent forbearance (26, 3%); (11) doing social work to help patients (26, 3%); (12) travelling (22, 2%); (13) eating (20, 2%); (14) meditation and yoga (8, 1%); (15) smoking and taking other sedative drugs (6, 1%) (Additional file 1: Table S6). Majority of research participants (817, 82%) thinks that their coping strategies effectively reduce stress, but 18% (173) thinks it does not work. The respondents also provide reasons for the failure to reduce stress. Listed reasons as reported were: (1) coping strategies are not enough to reduce stress (119, 12%); (2) anxiety to complete large course in short time (41, 4%); (3) lack of ability or self-confidence to cope with stress (13, 2%). Majority of participants of the study do not want to change their coping strategies however, 27% (270) were predispose to adopt new coping strategies. Listed new strategies were: (1) better planning with patience to solve problem and reduce stress (76, 8%); (2) extensive discussion with family and friends about the causes of stress (85, 8%); (3) discussion with teachers and seniors to resolve the problem (95, 9%); (4) follow a mentor to lead stress-less and successful life (8, 1%); (5) choose less stressful profession (6, 1%).

## Discussion

The response rate was about 73%. This finding is similar with a number research reports [29, 30]. Forty-nine percent of the research participants reported that their concentration remains the same, contrasting with other study findings [21, 31]. 41% of students enjoyed daily activities which is similar to the findings of previous researches [25, 31]. In addition, 32% maintained adequate sleep; 37% could overcome difficulties. Similar, results were also reported in other surveys [25, 31]. In contrast, only 26% research participants felt they were not loosing confidence; 26% felt unhappy and depressed which differ from the study outcome of other appraisals [9, 21, 32]. Similarly, 53% were capable of making good decisions; 55% were reasonably happy; 52% do not feel they are worthless. These findings are similar to the outcome of another study report [10].

University responsibilities are different and more demanding than high school level. Parent involvement is reduced and students live in dormitories during University sessions. Therefore, stress increases in many ‘tertiary’ level educations because of newly added responsibilities [25, 33, 34]. The current study indicated that the overall prevalence of stress is higher than a similar study conducted in Malaysia [35] and lower than other study in Southeastern Asia [36] but almost similar with other studies from developing countries [11, 25]. A number of reports concluded that positive emotional disorders exist in medical students as like in the current study findings [37, 38]. Medical students in public institution suffer more stress than their counterparts in private institutions. There was no significant difference between genders. Today women are at par with men in academic and professional achievements. Finally, Year-IV medical students are more stressed than Year-III. This can be attributed to the 2nd professional examination at the end of Year-IV. This study agrees with the previous finding that academic stress is the top of the list [11, 25, 35, 36]. The coping strategies of Bangladeshi medical students were different from those reported by other studies [11, 25, 35, 36, 39]. The different coping mechanism for stress reported by the students’ includes sharing any issues with family and friends (14%); watching television and movies (13%). These findings agree with the outcome of other studies [11, 31, 40]. In addition, self-counselling (12%), praying (12%), taking rest and sleep (9%), singing and listening songs (9%) are comparable to result of other researches [2, 9, 10]. However, 82% stated that coping mechanism is working for them; while, the rest 18% it is not. Comparable outcome were obtained in other surveys [41, 42]. Academic-stress was reported to be the most stressful by Bangladeshi medical students. Therefore, relevant authorities need to address this issue in order to produce high-quality professional and holistic medical doctors for the country [43–47]. Limitation of the Study: This is a cross sectional study with its own inherent limitation. Therefore this research only generates a snap shot of the whole miseries. The limited coverage cannot validity study the negative impact of stress on medical students.

## Conclusions

Both public and private medical schools students of Bangladesh suffered from stress. Medical students in public institution were reported to suffer more than private. Academic stress was found on the top of the list of the cause of stress. This study failed to detect any significant gender and religion related differences in the level of stress. However, statistically significant difference was observed between different cohort of students. The results of the current study will serve as baseline data for further in-depth prospective state sponsored study. Relevant authorities must take more initiatives and support research to develop proper policies for medical education and thus produce high-quality medical doctors who will serve Bangladesh and rest of world.

## Additional files

Additional file 1: Contains Tables 1–6. **Table 1**. Plan for Medical Curriculum in Bangladesh 2002. **Table 2**. Demographic information of the Study Participants. **Table 3**. Showing the result of GHQ-12. **Table 4**. Stressors (identified by the Medical Student Stressor Questionnaire) ranked by mean degree of stress perceived by medical students. **Table 5**. Differences Mean Scores of GHQ Based on Type Of Universities, Gender & Year Of Study. (n = 536). **Table 6**. Coping Strategies of the Study Participants. Additional file 2: Contain Stress Survey Questionnaire for Medical Students of Bangladesh which combines Sections A–D. Section A: Socio-Demographic Characteristics. Section B: General Health Question 12 (GHQ12). Section D: Coping Strategies. Section C: The Medical Student Stressor Questionnaire (MSSQ).

## Abbreviations

BDI: Beck’s depression inventory
GHQ: general health questionnaire
SCL: symptom check-list
DMC: Dhaka Medical College
SSMC: Sir Salimullah Medical College
EMC: Eastern Medical College
GMC: Gazi Medical College
CMC: Central Medical College
ZHSMC: ZH Sikder Medical College
UAMC: Uttara Adhunik Medical College
SMAMC: Sahid Monsur Ali Medical College
MSSQ: medical students stressor questionnaire
