# Supplementary material for: Prevalence of stress among medical students: a comparative study between public and private medical schools in Bangladesh
Source: BMC Res Notes. 2015 Jul 30;8:327. doi: 10.1186/s13104-015-1295-5 (PMC4520268; doi:10.1186/s13104-015-1295-5)
Supplement: Additional file 1: — Contains Tables 1–6. Table 1. Plan for Medical Curriculum in Bangladesh 2002. Table 2. Demographic information of the Study Participants. Table 3. Showing the result of GHQ-12. Table 4. Stressors (identified by the Medical Student Stressor Questionnaire) ranked by mean degree of stress perceived by medical students. Table 5. Differences Mean Scores of GHQ Based on Type Of Universities, Gender & Year Of Study. (n = 536). Table 6. Coping Strategies of the Study Participants. [file 13104_2015_1295_MOESM1_ESM.doc]

**Additional File 1:** Combines additional file tables 1-6. Table 1: Plan for Medical Curriculum in Bangladesh 2002. Table 2: Demographic information of the Study Participants. Table 3: Showing the result of GHQ-12. Table 4: Stressors (identified by the Medical Student Stressor Questionnaire) ranked by mean degree of stress perceived by medical students. Table 5: Differences Mean Scores of GHQ Based on Type Of Universities, Gender & Year Of Study. (n=536). Table 6: Coping Strategies of the Study Participants.

Table 1: Plan for Medical Curriculum in Bangladesh 2002

| **Phases** | **Phase I** | | **Phase II** | | **Phase III** |
| --- | --- | --- | --- | --- | --- |
| Duration | I ½ years | | 2 years | | I ½ years |
|  | 1^st^ year | 2^nd^ year | 3^rd^ year | 4^th^ year | 5^th^ year |
| Content | Anatomy  Physiology  Biochemistry | | Pharmacology and therapeutics  Forensic Medicine and  Toxicology  Pathology  Microbiology  Community Medicine | | Medicine  Surgery  Obstetrics and Gynaecology |
| Assessment | Formative assessment and  1^st^ Prof. Exam. at the end of 1½ years | | Formative assessment and  2^nd^ Prof. Exam. at the end of year 3½ years | | Formative assessment and  Final Prof.  at the end of year 5 |

Note: Pass mark is 60% in each written, oral and practical/clinical examination There are two professional examinations each year, January and July 10% marks of written summative examination comes from formative assessment

Table 2: Demographic information of the Study Participants

| Serial  Number | Items | | Frequency (%) |
| --- | --- | --- | --- |
| 1 | Gender | Male | 357 (36) |
|  |  | Female | 633 (64) |
| 2 | Year of Study | Year III | 579 (58) |
|  |  | Year IV | 411 (42) |
| 3 | Religion | Islam | 824 (83.2) |
|  |  | Hindu | 160 (16.2) |
|  |  | Christian | 2 (0.2) |
|  |  | others | 4 (0.4) |
| 4 | Marital Status | Single | 968 (98) |
|  |  | Married | 22 (2) |
| 5 | Accommodation | Hostel | 636 (64) |
|  |  | Parenteral Home | 255 (26) |
|  |  | Rental Home | 99 (10) |

**Table 3: Showing the result of GHQ-12**

| **Questions: Have you recently…** | **Responses** | **n (%)** | **Mean (SD)** | **Mean (SD)** |
| --- | --- | --- | --- | --- |
| been able to concentrate on whatever you are doing? | Better than usual | 145 (14.6) | 2.71 (0.81) | Positive items  2.74 (0.53) |
|  | Same as usual | 488 (49.3) |  |  |
|  | Less than usual | 277 (28.0) |  |  |
|  | Much less than usual | 80 (8.1) |  |  |
| felt that you are playing a useful part in things | More than usual | 127 (12.8) | 2.83 (0.71) |  |
|  | Same as usual | 616 (62.2) |  |  |
|  | Less useful than usual | 196 (19.8) |  |  |
|  | Much less useful | 51 (5.2) |  |  |
| felt capable of making decisions about things | More than usual | 185 (18.7) | 2.85 (0.81) |  |
|  | Same as usual | 547 (55.3) |  |  |
|  | Less capable than usual | 179 (18.1) |  |  |
|  | Much less capable | 79 (8.0) |  |  |
| been able to enjoy your normal day to day activities | More than usual | 100 (10.1) | 2.44 (0.89) |  |
|  | Same as usual | 408 (41.2) |  |  |
|  | Less than usual | 310 (31.3) |  |  |
|  | Much less than usual | 172 (17.4) |  |  |
| been able face up to your problems | More than usual | 195 (19.7) | 2.83 (0.82) |  |
|  | Same as usual | 499 (50.4) |  |  |
|  | Less able than usual | 230 (23.2) |  |  |
|  | Much less able | 66 (6.7) |  |  |
| been feeling reasonably happy, all things considered? | More than usual | 157 (15.9) | 2.78 (0.81) |  |
|  | Same as usual | 540 (54.5) |  |  |
|  | Less than usual | 212 (21.4) |  |  |
|  | Much less than usual | 81 (8.2) |  |  |
| lost much sleep over worry | Not at all | 319 (32.2) | 2.72 (1.12) | Negative items  2.79 (0.74) |
|  | No more than usual | 272 (27.5) |  |  |
|  | Rather than usual | 200 (20.2) |  |  |
|  | Much more than usual | 199 (20.1) |  |  |
| felt constantly under strain | Not at all | 219 (22.1) | 2.55 (1.06) |  |
|  | No more than usual | 313 (31.6) |  |  |
|  | Rather more than usual | 248 (25.1) |  |  |
|  | Much more than usual | 210 (21.2) |  |  |
| felt you couldn't overcome your difficulties | Not at all | 362 (36.6) | 2.91 (1.03) |  |
|  | No more than usual | 290 (29.3) |  |  |
|  | Rather more than usual | 222 (22.4) |  |  |
|  | Much more than usual | 116 (11.7) |  |  |
| been feeling unhappy and depressed | Not at all | 246 (24.8) | 2.53 (1.10) |  |
|  | No more than usual | 256 (25.9) |  |  |
|  | Rather more than usual | 260 (26.3) |  |  |
|  | Much more than usual | 228 (23.0) |  |  |
| been losing confidence in yourself | Not at all | 380 (38.4) | 2.88 (1.08) |  |
|  | No more than usual | 263 (26.6) |  |  |
|  | Rather more than usual | 199 (20.1) |  |  |
|  | Much more than usual | 148 (14.9) |  |  |
| been thinking of yourself as a worthless person | Not at all | 518 (52.3) | 3.19 (1.01) |  |
|  | No more than usual | 234 (23.6) |  |  |
|  | Rather more than usual | 144 (14.5) |  |  |
|  | Much more than usual | 94 (9.5) |  |  |
| Total GHQ, mean (SD) | 2.77 (0.58) | | | |

Table 4: Stressors (identified by the Medical Student Stressor Questionnaire) ranked by mean degree of stress perceived by medical students

| **Rank** | **Items** | ***Degree of stress, mean (SD)** |
| --- | --- | --- |
| **Causing nil to mild stress** | | |
| 1. | Talking to patient about personal problems | 0.81 (0.97) |
| **Causing mild to moderate stress** | | |
| 2. | Conflict with other students | 1.26 (1.22) |
| 3. | Verbal or physical abuse by other students | 1.47 (1.36) |
| 4. | Parental wish for you to study medicine | 1.15 (1.28) |
| 5. | Conflict with personnel | 1.24 (1.20) |
| 6. | Participation in class discussion | 1.13 (1.14) |
| 7. | Falling behind in reading schedule | 1.86 (1.30) |
| 8. | Participation in class presentation | 1.27 (1.19) |
| 9. | Learning context-full of competition | 1.74 (1.36) |
| 10. | Teacher lack of teaching skills | 1.70 (1.32) |
| 11. | Unable to answer question from patients | 1.66 (1.21) |
| 12. | Facing illness or death of patients | 1.73 (1.29) |
| 13. | Unable to answer the questions from the teachers | 1.83 (1.22) |
| 14. | Frequent interruption of my work by others | 1.86 (1.26) |
| 15. | Conflict with teachers | 1.76 (1.40) |
| 16. | Unwillingness to study medicine | 1.49 (1.38) |
| 17. | Not enough feedback from teachers | 1.78 (1.28) |
| 18. | Lack of recognition for work done | 1.79 (1.31) |
| 19. | Verbal or physical abuse by personnel | 1.62 (1.34) |
| 20. | Family responsibilities | 1.58 (1.39) |
| 21. | Quota system in examination | 1.50 (1.44) |
| 22. | Need to do well (self-expectation) | 1.82 (1.38) |
| 23. | Not enough study material | 1.24 (1.25) |
| 24. | Lack of guidance from teachers | 1.64 (1.35) |
| 25. | Feeling of incompetence | 1.65 (1.32) |
| 26. | Uncertainty of what is expected of me | 1.73 (1.25) |
| 27. | Not enough medical skill practice | 1.86 (1.25) |
| 28. | Lack of time for family and friends | 2.00 (1.32) |
| 29. | Inappropriate assignments | 1.62 (1.19) |
| 30. | Having difficulty understanding contents | 1.77 (1.18) |
| 31. | Poor motivation to learn | 1.88 (1.23) |
| 32. | Need to do well (imposed by others) | 1.84 (1.26) |
| 33. | Unjustified grading process | 1.90 (1.33) |
| 34. | Working with computers | 1.02 (1.21) |
| **Causing moderate to high stress** | | |
| 35. | Getting poor marks | 2.11 (1.35) |
| 36. | Verbal or physical abuse by teachers | 2.11 (1.38) |
| 37. | Heavy workload | 2.28 (1.36) |
| 38. | Lack of time to review what have been learnt | 2.17 (1.25) |
| 39. | Large amount of content to be learnt | 2.10 (1.30) |
| 40. | Test/ examinations | 2.39 (1.36) |

Degree of stress classification: 0 – 1.00 is ‘causing nil to mild stress’; 1.01 – 2.00 is ‘causing mild to moderate stress; 2.01 – 3.00 is ‘causing moderate to high stress’ and 3.01 – 4.00 is ‘causing high to severe stress’

Table 5: Differences Mean Scores of GHQ Based on Type Of Universities, Gender & Year Of Study. (n=536).

| Variables | | Mean | SD | 95% CI | *p* value* |
| --- | --- | --- | --- | --- | --- |
| Total GHQ | Public  (n = 337) | 2.84 | 0.59 | 0.03 | 0.005 |
|  | Private  (n = 653) | 2.73 | 0.57 | 0.19 |  |
|  | Male  (n = 357) | 2.81 | 0.57 | -0.00 | 0.060 |
|  | Female  (n = 633) | 2.74 | 0.59 | 0.15 |  |
|  | Year 3  (n = 579) | 2.81 | 0.58 | 0.04 | 0.004 |
|  | Year 4  (n = 411) | 2.70 | 0.58 | 0.04 |  |

Independent t-test

Table 6: Coping Strategies of the Study Participants

| Serial  Number | Strategy | Frequency (%) |
| --- | --- | --- |
|  | Share with Friends and Family | 135 (14) |
|  | Watch Television and Movies | 126 (13) |
|  | Self-Counselling | 123 (12) |
|  | Prayers | 117 (12) |
|  | Resting and Sleeping | 92 (9) |
|  | Listening and Singing Songs | 90 (9) |
|  | Net Surfing | 88 (9) |
|  | Reading Story Books and Dairy Writing | 69 (7) |
|  | Playing Games | 42 (4) |
|  | Remain Silence and Alone | 26 (3) |
|  | Social Work (Helping patients) | 26 (3) |
|  | Travelling | 22 (2) |
|  | Eating | 20 (2) |
|  | Meditation and Yoga | 8 (1) |
|  | Smoking and Taking Sedatives | 6 (1) |
