# Supplementary material for: Prevalence of stress among medical students: a comparative study between public and private medical schools in Bangladesh
Source: BMC Res Notes. 2015 Jul 30;8:327. doi: 10.1186/s13104-015-1295-5 (PMC4520268; doi:10.1186/s13104-015-1295-5)
Supplement: Additional file 2: — Contain Stress Survey Questionnaire for Medical Students of Bangladesh which combines Sections A–D. Section A: Socio-Demographic Characteristics. Section B: General Health Question 12 (GHQ12). Section D: Coping Strategies. Section C: The Medical Student Stressor Questionnaire (MSSQ). [file 13104_2015_1295_MOESM2_ESM.docx]

# Additional File 2: Combines Sections A-D. SECTION A: Socio-Demographic Characteristics. SECTION B: General Health Question 12 (GHQ12). SECTION D: Coping Strategies. SECTION C: The Medical Student Stressor Questionnaire (MSSQ).

# STRESS SURVEY QUESTIONNAIRE FOR MEDICAL STUDENTS OF BANGLADESH

**SECTION A: Socio-Demographic Characteristics** (Please answer by **Tick Mark** or **Writing** where appropriate)

**A1.** _________________________________ Medical College.

**A2**. Age: _________Years , Gender: Male/ Female

**A3.** Religion: Islam/ Hindu/ Christian/ Others (Please Specify) __________________

**A4.** Year of Study : First year/ Second year/ Third Year/ Fourth Year/ Fifth Year

**A5.** GPA: SSC _______; HSC _______; Average__________

**A6.** Marital Status: Single/ Married/ Others (Please Specify) _________________

**A7.** Residency: Hostel/ Parental home/ Rented Accommodation

**A8.** Motivation to Study Medicine: Own Interest/ Parental Influenced Choice/ Randomly choice

**A9.** Which ward posting you currently being: Surgery/ Medicine/ Gynae & Obs/ Paediatrics/ Orthopaedics/ Psychiatry/ Skin & VD/

Eye/ENT/ Radiology/ Anaesthesiology/ Others (Please Specify) _________________

# SECTION B: General Health Question 12 (GHQ12)

**Please read this carefully:** We should like to know if you have had any medical complaints, and how your health has been in general, currently and over the recent past few weeks maximum within a month. (Not those you had in the long past).

Please answer **ALL** the questions simply by **tick** the answer which you think most nearly applies to you.

| **Questions:** Have you recently… | **Answers:** | | | |
| --- | --- | --- | --- | --- |
| 1. been able to concentrate on whatever you are doing? | Better than usual | Same as usual | Less than usual | Much less than usual |
| 2. lost much sleep over worry? | Not at all | No more than usual | Rather more than usual | Much more than usual |
| 3. felt that you are playing a useful part in things? | More than usual | Same as usual | Less useful than usual | Much less useful |
| 4. felt capable of making decisions about things? | More than usual | Same as usual | Less capable than usual | Much less capable |
| 5. felt constantly under strain? | Not at all | No more than usual | Rather more than usual | Much more than usual |
| 6. felt you couldn’t overcome your difficulties? | Not at all | No more than usual | Rather more than usual | Much more than usual |
| 7. been able to enjoy your normal day to day activities? | More than usual | Same as usual | Less than usual | Much less than usual |
| 8. been able to face up to your problems? | More than usual | Same as usual | Less able than usual | Much less able |
| 9. been feeling unhappy and depressed? | Not at all | No more than usual | Rather more than usual | Much more than usual |
| 10. been losing confidence in yourself? | Not at all | No more than usual | Rather more than usual | Much more than usual |
| 11. been thinking of yourself as a worthless person? | Not at all | No more than usual | Rather more than usual | Much more than usual |
| 12. been feeling reasonably happy, all things considered? | More than usual | Same as usual | Less than usual | Much less than usual |

# SECTION C: The Medical Student Stressor Questionnaire (MSSQ). Please answer ALL the questions simply by TICK MARK.

| **Sl. No.** | **Items** | **Causing No**  **Stress**  **at All (0)** | **Causing**  **Mild**  **Stress (1)** | **Causing Moderate**  **Stress (2)** | **Causing**  **High**  **Stress (3)** | **Causing**  **Severe**  **Stress (4)** |
| --- | --- | --- | --- | --- | --- | --- |
| 1 D-I | Test/ Examinations |  |  |  |  |  |
| 2 D-IV | Talking to patient about personal problems |  |  |  |  |  |
| 3 D-II | Conflict with other students |  |  |  |  |  |
| 4 D-I | Quota system in examination |  |  |  |  |  |
| 5 D-II | Verbal or physical abuse by other students/s |  |  |  |  |  |
| 6 D-V | Parental wish for you to study medicine |  |  |  |  |  |
| 7 D-I | Need to do well (self-expectation) |  |  |  |  |  |
| 8 D-III | Not enough study material |  |  |  |  |  |
| 9 D-II | Conflict with personnel/s |  |  |  |  |  |
| 10 D-I | Heavy workload |  |  |  |  |  |
| 11 D-VI | Participation in class discussion |  |  |  |  |  |
| 12 D-I | Falling behind in reading schedule |  |  |  |  |  |
| 13 D-VI | Participation in class presentation |  |  |  |  |  |
| 14 D-III | Lack of guidance from teacher/s |  |  |  |  |  |
| 15 D-VI | Feeling of incompetence |  |  |  |  |  |
| 16 D-III | Uncertainty of what is expected of me |  |  |  |  |  |
| 17 D-I | Not enough medical skill practice |  |  |  |  |  |
| 18 D-IV | Lack of time for family and friends |  |  |  |  |  |
| 19 D-I | Learning context – full of competition |  |  |  |  |  |
| 20 D-III | Teacher – lack of teaching skills |  |  |  |  |  |
| 21 D-IV | Unable to answer question from patients |  |  |  |  |  |
| 22 D-III | Inappropriate assignments |  |  |  |  |  |
| 23 D-I | Having difficulty understanding contents |  |  |  |  |  |
| 24 D-IV | Facing illness or death of the patients |  |  |  |  |  |
| 25 D-I | Getting poor marks |  |  |  |  |  |
| 26 D-II | Poor motivation to learn |  |  |  |  |  |
| 27 D-I | Lack of time to review what have been learnt |  |  |  |  |  |
| 28 D-II | Verbal or physical abuse by teacher/s |  |  |  |  |  |
| 29 D-IV | Frequent interruption of my work by others |  |  |  |  |  |
| 30 D-I | Unable to answer questions from teachers |  |  |  |  |  |
| 31 D-II | Conflict with teacher/s |  |  |  |  |  |
| 32 D-V | Unwillingness to study medicine |  |  |  |  |  |
| 33 D-I | Large amount of content to be learnt |  |  |  |  |  |
| 34 D-VI | Need to do well (imposed by others) |  |  |  |  |  |
| 35 D-III | Not enough feedback from teacher/s |  |  |  |  |  |
| 36 D-I | Unjustified grading process |  |  |  |  |  |
| 37 D-III | Lack of recognition for work done |  |  |  |  |  |
| 38 D-IV | Working with computers |  |  |  |  |  |
| 39 D-II | Verbal or physical abuse by personnel/s |  |  |  |  |  |
| 40 D-V | Family responsibilities |  |  |  |  |  |

# SECTION D: Coping Strategies

Please answer all the questions below. Kindly write **ONE** answer for each question.

**D1.** What is the coping strategy that you practice most of the time?

**D2.** Do you think your coping strategy of stress is effective on reducing your stress? Yes/ No; If No, why?

**D3.** Would you like to change your coping strategy in future? Yes/ No; If yes, what is the coping strategy that you prefer?
